# Effectiveness and safety of low dose Rituximab as remission-maintenance treatment for patients with refractory idiopathic inflammatory myopathies: results of a retrospective study from a monocentric cohort

**DOI:** 10.1007/s10067-024-07079-z

**Published:** 2024-08-28

**Authors:** Anna Gamba, Roberto Depascale, Elisabetta Zanatta, Luana Ienna, Claudio Cruciani, Mariele Gatto, Margherita Zen, Andrea Doria, Luca Iaccarino

**Affiliations:** 1 Rheumatology Unit, Division of Rheumatology, Department of Medicine-DIMED, University of Padova, Via Giustiniani, 2, 35128 Padova, Italy; 2 Department of Cardiac, Thoracic, Vascular Sciences and Public Health, University of Padova, Padova, Italy; 3 Academic Rheumatology Centre, Department of Clinical and Biological Sciences, University of Turin, AO Mauriziano di Torino, Turin, Italy

**Keywords:** Inflammatory myopathies, Interstitial lung disease, Maintenance treatment, Rituximab

## Abstract

**Objective:**

Our aim was to assess efficacy and safety of Rituximab (RTX) in patients with refractory Idiopathic inflammatory myopathies (IIM) from a monocentric cohort. Thereafter, we evaluated the efficacy of a low-dose RTX regimen as a remission-maintenance therapy.

**Methods:**

We retrospectively evaluated a cohort of patients affected with IIM treated with RTX. All patients were refractory to glucocorticoids (GC) and at least one immunosuppressant. Two infusions of 1 g two weeks apart were considered as standard cycle of RTX, a single dose of 1 g every six months was deemed as a low-dose RTX regimen. Complete and partial response were defined according to physician’s judgment, laboratory and radiological features.

**Results:**

Thirty-six patients affected with IIM were enrolled. Eighteen patients (50%) required the use of RTX for muscular involvement, 6 (16.7%) for interstitial lung disease (ILD), 12 (33.3%) for both myositis and ILD. We observed complete response to RTX in 25 patients (69.4%), partial response in 7 (19.4%) and no response in 4 (11.1%), with an overall response of 88.8% (partial and complete response). From the subgroup of twenty-five patients that achieved a complete response, six were treated with a low dose maintenance therapy maintaining a complete response to RTX. Twenty-six patients who achieved a complete or partial response were able to decrease the mean daily GC dose. Infections were the major adverse events detected in our study.

**Conclusions:**

RTX shows favorable outcomes in refractory patients with IIM. A low-dose regimen of RTX appears to be effective in maintaining remission after induction with standard dose.

**Table:** 

**Key Points** • *The precise pathogenic mechanism of idiopathic inflammatory myopathies (IIM) remains elusive; however, a growing body of data support the autoimmune hypothesis. In this context, rituximab, a B cell-depleting agent, has emerged as a second-line therapeutic option in IIM.* • *Several studies have assessed It its effectiveness in refractory IIM patients.* • *Limited information exists on the use of Rituximab as maintenance therapy in patients who have achieved remission following induction therapy with Rituximab.*

## Introduction

The term “idiopathic inflammatory myopathies” (IIM) refers to a spectrum of diseases that encompasses dermatomyositis (DM), polymyositis (PM), inclusion body myositis (IBM), necrotizing autoimmune myopathy (NAM), and anti-synthetase syndrome [1].

The specific pathogenetic mechanism of IIM is still unknown; however, the good response to immunosuppressive drugs, the association with other autoimmune diseases and finding of specific autoantibodies in about 75% of patients with IIM [2–4], support the hypothesis of an autoimmune etiology. These systemic autoimmune disorders are characterized by damage to muscle leading to inflammation within the muscles and a reduction in skeletal muscle strength. The management of this condition can be challenging due to its tendency to affect areas beyond the muscles. These additional complications frequently involve skin, joints, lung, gastrointestinal systema and heart [5]. The lung involvement associated with ILD can dramatically worse the course of the disease, intensifying the overall health burden of patients. The substantial rise in mortality linked to ILD underscores its importance as a key factor in the prognosis and management of individuals with IIM.

In addition, treatment refractoriness is not uncommon in IIM. This situation is further complicated by the absence of universally accepted guidelines for treating IIM, which leaves physicians with uncertainties about the best approach to patient care. Moreover, IIM patients frequently face two concurrent issues: an active, ongoing disease process and accumulated organ damage from long-term illness. This combination of factors complicates treatment strategies and patient outcomes. Patients not only struggle with the acute manifestations of their active disease but also contend with the long-term consequences of organ damage that has built up over time.

In line with this, rituximab (RTX), a B cell depleting agent, has been used as a second-line therapeutic choice in IIM, despite a lack of classical phase III randomized controlled trials testing its efficacy *vs* placebo, based on one phase II randomized trial and a number of open label and observational studies or small case series, all suggesting the effectiveness of this treatment in refractory patients [6–11]. On the other hand, a few data are available on the use of RTX as a maintenance therapy in patients who achieved remission after induction therapy with RTX.

Therefore, the aim of this study was not only to assess the efficacy and safety of RTX in a monocentric cohort of patients affected with refractory IIM, but also to evaluate the effectiveness of low-dose RTX as a remission-maintenance therapy in a subgroup of remitted patients.

## Patients and methods

We reviewed all consecutive patients affected with IIM prospectively followed in our Center from January 1st 2008 to June 30th 2023; demographic, clinic, laboratory and therapeutic data were reported in an ad hoc database and retrospectively evaluated for this study. The study was carried out in the Padova University Hospital, which is a third level referral hospital for North East Italy for autoimmune rheumatic diseases.

### Inclusion and exclusion criteria

We included patients according to the following inclusion criteria:

- Intravenous treatment with RTX, at least 1 g;
- Fulfilling at least one of the following classification criteria for IIM: Bohan & Peter [12, 13], ENMC [14] and 2017 EULAR/ACR criteria [15].
- Patients with refractory disease, defined as no response to glucocorticoid (GC) and at least one immunosuppressive drug, according to the physician judgment, before starting RTX

Exclusion criteria were the following:

- absence of at least one clinic evaluation within six months to one year after RTX administration
- inadequate clinical data availability

### Rituximab schedule

Two infusions of 1 g two weeks apart were considered as standard dose of RTX.

A single dose of 1 g every six months was defined as a low-dose RTX.

When RTX was administered every six months it was defined as a “scheduled therapy”, while when it was given during disease flares, it was considered “on-demand”.

Before each infusion of RTX, patients received an intravenous premedication with 80 mg of methylprednisolone, chlorpheniramine and acetaminophen.

From 2018, in our center it was decided to offer a maintenance therapy option to all IIM patients who had achieved the complete response to RTX.

### Response to Rituximab

Treatment outcomes were defined as follows:

- *Patients with muscular involvement:* partial response was defined as decrease of at least 30% of Creatine Phosphokinase (CK) serum levels and increase of 15% in Manual Muscle Test-8 score (MMT-8) or MMT-8 > 140/150, while a complete response was defined as normalization of CK levels (≤ 180 U/L) and increase of at least 20% in MMT-8 score or MMT-8 145/150.
- *Patients with pulmonary involvement:* partial and complete response were based on pneumologist judgment according to lung function tests and/or radiographic changes on high resolution chest computed tomography scan (HRCT) performed before and after RTX treatment. Patients with lung involvement underwent a HRCT every year and a pulmonary function test every 6 months. DLCO testing was incorporated as part of each spirometry examination.
- *Patients with pulmonary and muscle involvement:* complete response was achieved when criteria for complete response were obtained in both manifestations, otherwise it was considered a partial response.
- *Patients with skin and joint involvement:* partial and complete responses were based on physician judgment.

### Relapse

Relapse of IIM was defined by physician judgement based on worsening of the underlining clinical condition and the need to change or increase immunosuppressive treatment.

### Safety

Adverse event (AE) was defined as follow: “any untoward medical occurrence in a patient treated with a pharmaceutical product which does not necessarily have a causal relationship with this treatment”. The AE was defined as severe (SAE) if hospitalization was required.

In addition, being infusion-related-reactions (IRRs) and infections the major side effect of RTX according to literature data [8, 9], we defined as adverse event of special interest (AESI) the occurrence of an infectious disease or an IRR. We considered AESI occurred within one year after the last RTX infusion, since CD20-expressing B-cells levels usually take 6–12 months to return to pretreatment values [10].

## Results

### Patients characteristics

As reported in Table 1, 36 patients were included, 25 (69.4%) were female. Of them 15 (41.7%) had PM, 13 (36.1%) antisynthetase syndrome, 6 (16.7%) DM, 1 (2.8%) IBM, and 1 (2.8%) NAM.

**Table 1:** Demographic and clinical characteristics of IIM patients requiring RTX for muscular involvement, lung involvement, and muscular plus lung involvement

	Muscular involvement (*N* = 18)	ILD (*N* = 6)	ILD + Muscular involvement (*N* = 12)
Sex (F/M)	9/9	5/1	11/1
Age at disease onset, years (mean ± SD)	50 (± 26)	44 (± 9)	49 (± 11)
Lag time between diagnosis and RTX, years (mean ± SD)	3.5 (± 2.5)	6.0 (± 9.0)	6.0 (± 5.8)
Concomitant therapies*	PDN (*N* = 18) 100% MTX (*N* = 11) 61.1% MMF (*N* = 4) 22.2% IVIg (*N* = 2) 11.1% HCQ (*N* = 1) 5.5%	PDN (*N* = 4) 66.7% MMF (*N* = 4) 66.7% LEF (*N* = 1) 16.7% MTX (*N* = 1) 16.7% HCQ (*N* = 1) 16.7%	PDN (*N* = 11) 91.7% MTX (*N* = 6) 50.0% MMF (*N* = 4) 33.3% IVIg (*N* = 3) 25.0% AZA (*N* = 2) 16.7% HCQ (*N* = 2) 16.7% CsA (*N* = 1) 8.3%
Other disease manifestations	Skin involvement (*N* = 2) 11.1%	Arthritis (*N* = 5) 83.3%	Skin involvement (*N* = 3) 25.0% Arthritis (*N* = 2) 16.7%
ILD subtype	-	UIP (*N* = 2) 33.5% NSIP (*N* = 3) 50.0% OP (*N* = 1) 16.7%	UIP (*N* = 4) 33.3%, NSIP (*N* = 8) 66.7%
Number of RTX cycles (mean ± SD)	1.9 (± 1.4)	5.0 (± 2.4)	3.5 (± 3.3)
Follow up (months)	44 (± 38)	103 (± 40)	78 (± 51)
CPK at baseline (U/L) (mean ± SD)	1842 (± 300)	280 (± 350)	1687 (± 290)
MMT-8 at baseline (mean ± SD)	120.0 (± 22.0)	135.0(± 23.9)	122.0 (± 21.8)
PDN equivalent before RTX (mg/day) (mean ± SD)	15.7 (± 11.0)	15.3 (± 8.5)	11.6 (± 6.2)
PDN equivalent after RTX (mg/day) (mean ± SD)	8.2 (± 6.0)	5.8 (± 7.4)	4.0 (± 2.2)

*AZA* Azathioprine; *CPK* Creatine phosphokinase; *CsA* Cyclosporine A; *ILD* Interstitial lung disease; *IVIg* Intravenous immunoglobulin; *HCQ* Hydroxicloroquine; *LEF* Leflunomide; *MMF* Mycophenolate mofetil; *MMT-8* Manual muscle test 8; *MTX* Methotrexate; *NSIP* Nonspecific interstitial pneumonia; *OP* Organizing pneumonia; *PDN* Prednisone equivalent; *RTX* Rituximab; *UIP* Usual interstitial pneumonia.* the total of the percentages for various therapies exceeds 100% because many patients in our study were receiving a combination of treatments

Autoantibody positivity was found in 26 patients (72.2%), anti-Jo1 in 12 (33.3%), anti-SSA in 9 (25.0%), anti-SRP in 5 (13.9%), anti-PM/Scl75 in 5 (5.9%), anti-Mi2 in 5 (5.9%), anti-PL7 in 1 (2.8%), anti-PL12 in 1 (2.8%), anti-SSB in 1 (2.8%), anti-TIF1-γ in 1 (2.8%), anti-Ku in 1 (2.8%), and anti Sp100 in 1 (2.8%). Autoantibodies were negative in 10 cases (27.8%).

Previous treatments before RTX were: GCs in 36 patients (100%), methotrexate in 26 (72.2%), mycophenolate mofetil in 18 (50.0%), intravenous immunoglobulins (IVIg) in 10 (27.8%), azathioprine in 8 (22.2%), hydroxychloroquine in 5 (13.9%), cyclosporine A in 5 (13.9%) leflunomide in 4 (11.1%), tacrolimus in 1 (2.8%), and cyclophosphamide in 1 (2.8%). Before RTX initiation, GC and one immunosuppressive drug were previously used in 14 cases (38.8%), GCs and two immunosuppressants in 9 cases (25.0%) and GC and more than two immunosuppressants in 13 cases (36.1%). Mean lag-time between symptoms onset and first cycle of standard-dose RTX was 67 months (± 69 months).

Eighteen patients (50.0%), required the use of RTX for muscular involvement, 6 (16.7%) for ILD, 12 (33.3%) for both muscular and lung disease (Table 1). Of them, 7 (19.4%) had also arthritis and 5 (13.9%) had skin manifestations.

At RTX initiation, 33 patients (91.7%) were taking GCs at a mean prednisone dose equivalent of 14.85 mg/day, 18 (50.0%) methotrexate, 12 (33.3%) mycophenolate mofetil, 5 (13.9%) IVIG, 4 (11.1%) hydroxychloroquine, 2 (5.5%) azathioprine, 1 (2.8%) leflunomide and 1 (2.8%) cyclosporine. Overall, patients received a minimum of one cycle and a maximum of ten cycles (full and low dose) of RTX, with an average of 3.3 ± 3.1 cycles. Patients had a mean follow-up of 65 ± 48 months.

### Efficacy of rituximab

**Fig. 1:**
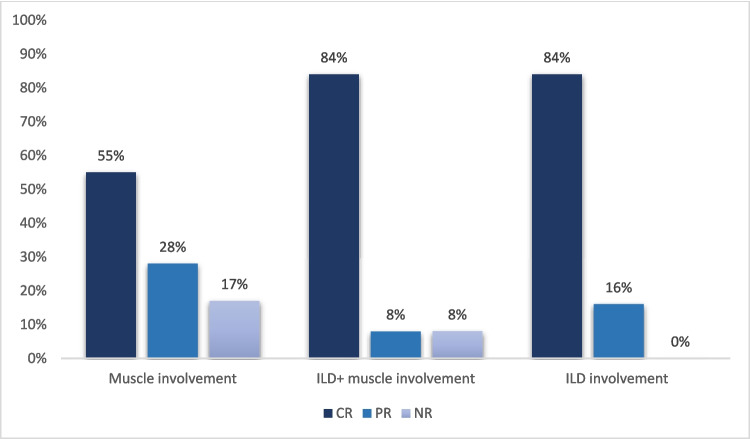
Response to RTX. CR: complete response; ILD: interstitial lung disease; NR: no response; PR: partial response

We observed a complete response to RTX in 25 patients (69.4%), partial response in 7 (19.4%), no-response in 4 (11.1%); therefore, we observed an overall response (complete and partial) of 88.8%. As shown in Fig. 1, we found a similar proportion of response to RTX in each group of patients with different disease manifestations.

The group of patients who achieved a complete response to RTX had a longer mean interval of 73.2 months (± 78 months) from diagnosis to treatment initiation. In contrast, the non-responder group had a shorter average interval of 54 months (± 34 months) between diagnosis and the start of RTX therapy; however, the difference was non statistically significant.

**Fig. 2:**
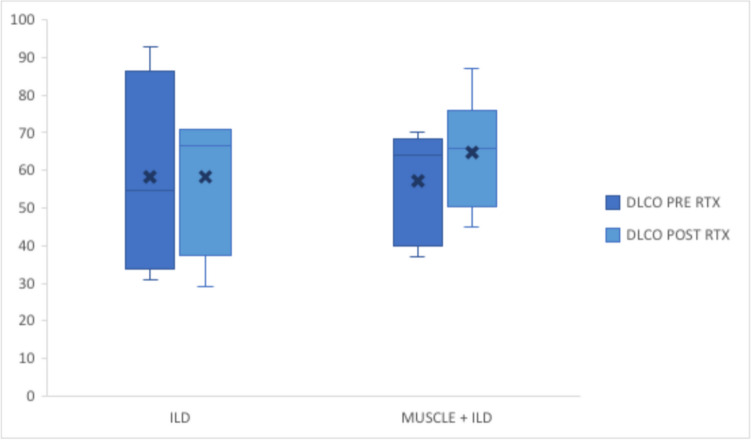
DLCO before and after RTX treatment in patients with lung and muscle + lung involvement DLCO: diffusing capacity of the lungs for carbon monoxide; ILD: interstitial lung disease, RTX: rituximab. X = average. *P*-value: 0.2

In Fig. 2 the variation of Diffusion Lung Carbon Monoxide (DLCO) before and after RTX in patients treated for pulmonary involvement is reported. Although not statistically significant, we observed an improvement in respiratory function.

Finally, we observed a complete or partial response in all patients with skin and joint manifestations.

**Fig. 3:**
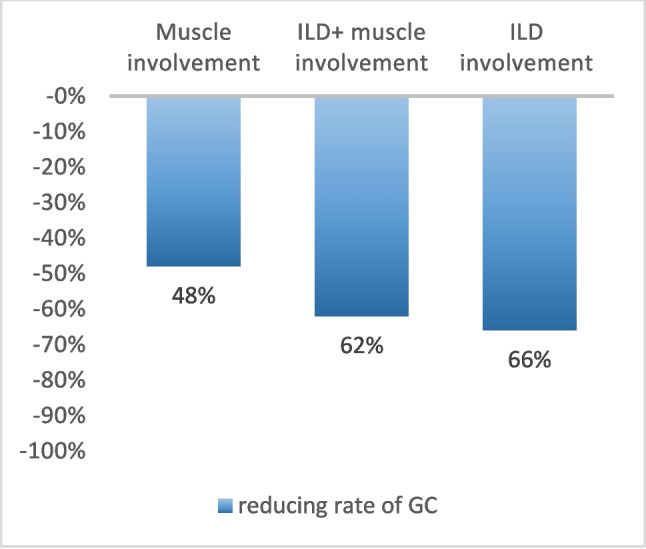
Decrease rate of oral daily prednisone after one year of RTX treatment. GC: glucocorticoids; ILD: interstitial lung disease

Twenty-six patients (81.2%) who achieved a complete or partial response were also able to decrease the daily GC dose from 15.0 ± 9.5 to 6.6 ± 5.8 mg/day. As shown in Fig. 3, the greatest decrease was observed in patients with lung involvement. Three patients (8.3%) were able to completely withdraw GCs.

### Low dose Rituximab

From the subgroup of twenty-five patients who achieved a complete response with standard dose, six were treated with a low dose maintenance therapy (Table 2).

**Table 2:** Demographic characteristics and response to rituximab in patients treated with low dose RTX

	Pt1	Pt2	Pt3	Pt4	Pt5	Pt6
Sex	M	F	M	F	F	M
Diagnosis	PM	ASS	ASS	PM	PM	ASS
Autoantibody	Anti-SRP	Anti-Jo1	Anti-Jo1	Anti-SRP	Anti-SRP	Anti-Jo1
Manifestation requiring RTX	ILD	ILD	Myositis	Myositis + ILD	Myositis + ILD	Myositis
Previous therapies	PDN, MMF	PDN, LEF, AZA, MMF, MTX	PDN, MTX	PDN, MTX	PDN, MTX, AZA, IVIg	PDN, MTX
Concomitant therapies	PDN, MMF	PDN, MMF	PDN; MTX	PDN, MTX	PDN, MTX, IVIg	PDN, MTX
N° cycle of standard dose RTX	2	2	4	6	6	2
N° cycle low dose RTX	7	6	3	4	5	1
PDN eq before RTX (mg/day)	18.75	12.5	12.5	6.25	31.25	25
PDN eq after RTX (mg/day)	0	2.5	2.5	2.5	6.25	10
N° of flares before standard dose RTX	1	3	2	1	4	1
N° of flares during standard dose RTX	0	0	0	0	0	0
N° of flares during low dose RTX	0	0	0	0	0	0
Maintained response	Yes	Yes	Yes	Yes	Yes	Yes
Follow up (months)	60	81	77	133	125	29

*ASS* Antisynthetase syndrome; *AZA* Azathioprine; *EQ* Equivalent; *ILD* Interstitial lung disease; *IVIg* Intravenous immunoglobulin; *LEF* Leflunomide; *MMF* Mycophenolate mofetil; *MTX* Methotrexate; *PDN* Prednisone equivalent; *PM* Polymyositis; *Pt* Patient; *RTX* Rituximab

Patients were treated with a mean number of 3.6 ± 1.9 RTX cycles (standard dose) before switching to low dose RTX.

All of them maintained a complete response to RTX and no flares were observed during the follow-up.

Finally, daily GC dose decreased in all patients treated with low dose regimen after remission, from 17.7 (± 9.1) to 5.0 (± 7.1) mg/day at the end of follow-up, and one patient was able to withdraw GCs.

In addition, the CD19 lymphocyte counts remained 0% during maintenance therapy. After the full-dose treatment, five out of six patients developed a mild IgM deficiency (0.30 g/l ± 0.05 g/l, with normal value > 0.4 g/l), which persisted even during the low-dose regimen. IgG and IgA levels have always been within normal ranges.

### Disease relapses

From the subgroup of 19 patients that achieved and maintained a complete response with RTX standard dose, 12 (63.2%) were treated with “on-demand” strategy and 7 (36.8%) with scheduled RTX, based on physician judgment. In the group treated with on-demand strategy we observed a disease flare in 2 patients (16.7%) within 12 months after the last RTX infusion. One out 7 (14.2%) patients treated with scheduled RTX, had a disease flare within 1 year after the last RTX administration.

### Safety

Nine out of 36 patients (25%) had an AESI within the 12 months following RTX infusions, requiring hospitalization in 6 cases (66.6%).

Two patients had pneumonia, including one pulmonary aspergillosis, two had Covid-19, two had a soft tissue infection, one had varicella zoster virus (VZV) infection and panniculitis, one had VZV infection, and one had a brain abscess due to toxoplasma infection. Notably, only 1 out of 6 patients on low dose maintenance RTX experienced an AESI (16.6%) compared to 8 out of 19 patients kept on standard dose (42.1%).

SAE occurred in six cases (16.7%), all in patients treated with standard maintenance dose.

No deaths occurred during the course of treatment and during follow-up (Table 3).

**Table 3:** AESI in patients treated with only standard dose RTX (Pt. 1–6) and in patients treated with both standard and low dose RTX (Pt 7–8)

Pt	AESI	Hospitalization (Yes/No)	RTX regimen during AESI
1	Bacterial pneumoniae	Yes	Full dose
2	Panniculitis	No	Full dose
3	Panniculitis	Yes	Full dose
4	Toxoplasma brain abscess	Yes	Full dose
5	Covid-19	Yes	Full dose
6	Reactivation of latent VZV	No	Full dose
7	Pulmonary aspergillosis	Yes	Full dose
8	Reactivation of latent VZV and panniculitis	No	Full dose
9	Reactivation of latent VZV and Covid-19	Yes	Low dose

*AESI* Adverse events of special interest; *Pt* Patient; *VZV* Varicella zoster virus

## Discussion

In our cohort, we assessed the response to RTX in refractory patients with IIM. Due to the rarity of the disease and the heterogeneity of organ involvement, there are no shared guidelines on the therapeutic approach in patients with IIM and especially in case of refractory disease. On the other hand, the unique randomized controlled trial of RTX in DM and PM available in literature showed 83% of patients achieving definition of improvement after initiating treatment with RTX [7]. Other observational studies or cases series reported similar results [8–11]. Data from our monocentric cohort are in line with those reported in literature, being RTX effective in the majority (88.8%) of multi-drug refractory patients; notably, most of them (68.4%) achieved a complete response.

We categorized patients according to the prevalent organ involvement which required the treatment. As expected, RTX was only prescribed in patients with prevalent muscular and lung involvement; joint or skin disease was never the prevalent manifestation requiring RTX. However, being concomitant with muscular and lung involvement in some cases, it was possible to demonstrate the efficacy of RTX also in these manifestations.

We observed a similar good response rate in both patients with lung and/or muscle involvement and this finding has considerable impact, since ILD is one of the major causes of morbidity and mortality in IIM patients [16, 17].

In addition, a GC-sparing effect of RTX was observed. Considering the entire follow-up, the great majority of patients who responded to RTX was able to decrease daily dosage of GCs and some of them were able to withdraw the drug. Our results are in line with other literature data [6, 18, 19]. Notably, the decrease in the intake of GC is one of the main goals in the modern management of patients with systemic rheumatic diseases [20]. Although it has not been directly demonstrated in patients with IIM, it is well known that a prolonged use of GC is one of the main causes of damage accrual [2, 21–23].

Furthermore, we assessed the efficacy of a low dose of RTX as maintenance therapy in a group of patients who had already achieved a complete response to treatment. Efficacy of low dose RTX has been recently reported also by Janardana et al. [19]. The Authors treated 14 patients with IIM with a low dose regimen of RTX (500 mg two doses two weeks apart) as induction therapy and found a similar efficacy of 28 patients treated with standard dose (1 g two doses 2 weeks apart). Differently from this study we used a different “low dose” scheme which consisted in a unique intravenous infusion of 1 g RTX, thus minimizing the patient’s need to go to the hospital. Moreover, in our study the low dose was used as maintenance therapy, and not as induction therapy; we showed that all patients treated with this approach maintained the remission achieved with the previous standard dose; on the other hand, this approach also guaranteed the possibility to further spare GCs. Finally, although heterogeneous, the length of follow-up was longer than one year in all cases, suggesting the beneficial long-term effect of this approach.

RTX showed a safety profile in line with what reported in literature, being infections the major AESI observed in this study. The possibility that in clinical practice a patient affected with IIM could develop a severe infection when treated with anti-CD20 therapy, or more in general with immunosuppressants, should be always kept in mind [24]. Interestingly, in our study only one SAE occurred following treatment with a low dose of RTX. Nevertheless, in our study, the lack of a control population does not allow to establish a casual association between infections and RTX administration.

### Strengths and limitations

This study is based on data collected from real-world clinical practice with a long period of follow-up. Furthermore, we had one of the largest sample size reported in literature for a monocentric cohort. Finally, by performing a single-center study we were able to ensure uniformity of treatment for all patients.

Main limitations include the absence of a control group and the retrospective design of the study which limit the full availability of clinical data.

## Conclusions

In our study RTX was effective in a great majority of refractory patients affected with IIM. Low dose RTX maintenance therapy seems able to maintain the remission achieved with the previous standard RTX dose. Infections are the main AESI observed in patients treated with both standard and low dose RTX maintenance therapy. Future studies could evaluate the minimal duration of the maintenance regimen able to guarantee prolonged remission and which markers are most reliable in monitoring disease activity, to allow personalization of treatment.

## Data Availability

Not applicable.
